# A working model for CCN3 C‐terminal domain‐mediated transcriptional modulation of the plasminogen activation system

**DOI:** 10.1002/ccs3.70083

**Published:** 2026-05-19

**Authors:** Bernard Perbal

**Affiliations:** ^1^ International CCN Society Nice France

**Keywords:** CCN C‐terminal domain, CCN proteins, CCN3, CCN5, moonlighting proteins, nuclear proteoforms, plaminogen inhibitor (PAI‐2), plasminogen, PolII, rpb7, transcription factors, tumor phenotype, wound healing

## Abstract

Over the past 2 decades, major advances have contributed to the elucidation of the structural and biochemical bases underlying the biological activities attributed to CCN proteins. The concept that CCN proteins exhibit bifunctional “moonlighting” properties in both the extracellular matrix (ECM) and the cell nucleus has recently emerged. CCN proteins participate in dual signaling processes, integrating combinatorial interactions with regulatory ligands, cell surface receptors or associated co‐receptors such as heparan sulfate proteoglycans (HSPGs), LRPs, TrkA, Notch, integrins, BMP‐4, TGF‐β, and FGFR2, as well as transcription factors in the nuclear compartment. In this manuscript, we propose an exploratory integrative model that brings together previously unassociated observations into a coherent framework. In this model, the C‐terminal module, present in all CCN proteins except CCN5, is proposed to direct the formation of homo‐ and heterodimers, which constitute a fundamental level of transcriptional regulation.

## Graphical abstract

Plasminogen is a circulating zymogen that is converted into active plasmin by plasminogen activators such as tissue plasminogen activator (tPA) and urokinase‐type PA (uPA). This conversion is regulated by plasminogen activator inhibitors (PAI‐1, PAI‐2) and by inhibitors of plasmin itself, maintaining a fine balance between activation and inhibition of the fibrinolytic system; excessive or reduced activity can have pathological consequences (thromboembolism, bleeding disorders). In this diagram, the C‐terminal (CT) domain of CCN3, by interacting with components of the system, blocks the activity of the plasminogen activator (red cross), thereby influencing plasmin production and fibrinolysis.

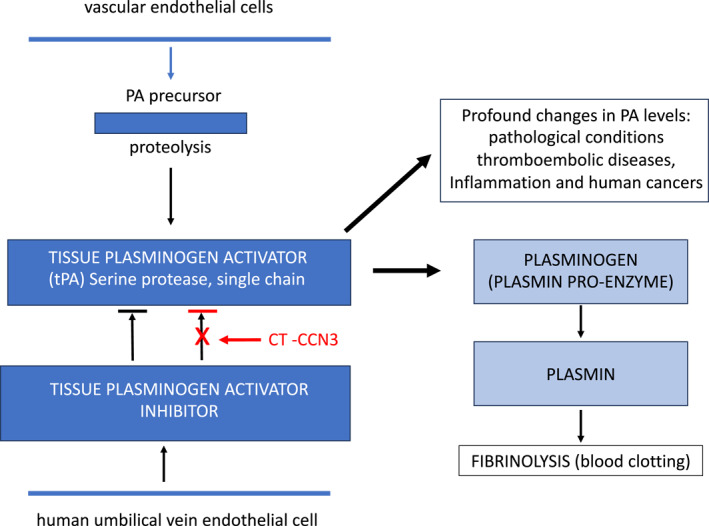

## 1 INTRODUCTION

The CCN proteins constitute a family of secreted regulatory molecules that participate in the regulation of several aspects of cell proliferation and differentiation, including migration, mitogenesis, wound healing, chondrogenesis and apoptosis. They are considered as major regulators of communication with the extracellular environment in normal and pathological contexts such as angiogenesis and tumorigenesis.

Although they share a highly conserved modular organization,^1^, ^2^, ^3^, ^4^, ^5^, ^6^, ^7^ accumulating evidence indicates that individual CCN members exert distinct and sometimes opposing biological functions. Understanding how structurally related proteins generate such functional diversity remains an important question in CCN biology.

The genes encoding CCN proteins were originally identified in different experimental contexts. Two founding members, CYR61 and CTGF, were first described as immediate‐early genes rapidly induced following serum stimulation of quiescent cells re‐entering the G1 phase of the cell cycle, without requiring de novo protein synthesis.^2^, ^3^ In contrast, NOV (CCN3) was characterized as a negative regulator of cell proliferation preferentially expressed in quiescent cells.^2^, ^3^ The overexpression of CCN3 resulted in a decrease of VCAM‐1 expression whereas knock down of CCN3 increased the expression of VCAM‐1 via its effects on NF‐KB nuclear translocation and DNA binding to the VCAM promoter^8^ Additional members were later identified through studies of Wnt‐1–transformed mammary epithelial cells, leading to the isolation of the WISP genes. Despite their distinct discovery pathways, these proteins were subsequently recognized to share strong sequence similarities and conserved structural features.^9^

These observations led to the definition of the CCN family, now composed of six members (CCN1–CCN6), whose nomenclature was later standardized by the HUGO Gene Nomenclature Committee.^10^

In this review, we focus on observations suggesting that the CCN3 protein also participates in the regulation of transcription of the plasminogen activation system, thereby highlighting mechanistic aspects of its biological functions.

## 2 STRUCTURAL ORGANIZATION AND BIOCHEMICAL DIVERSITY

All CCN proteins share a conserved primary structure and modular organization composed of three N‐terminal domains, IGFBP, VWC, and TSP1, preceded by a signal peptide that directs their secretion.^2^, ^4^, ^5^, ^6^, ^7^

Importantly, the presence of similar structural motifs should not be interpreted as evidence for identical functions. As in many modular proteins, the biological properties of CCN molecules likely emerge from the combined and context‐dependent activities of their individual domains.

Although the CCN modular organization and primary sequences have been conserved for more than 500 million years, the acquisition of a fourth C‐terminal (CT) module occurred later during evolution, approximately 150 million years ago, and has remained absent in CCN5 throughout evolution.^11^, ^12^

This distinctive feature raises important questions regarding the functional contribution of the CT domain to CCN protein activity.^13^ It has traditionally been interpreted as contributing to a reduction or modification of canonical CCN family activities. In contrast, the exploratory integrative model presented here suggests that the lack of CT incorporation may represent a key determinant of CCN5 functional divergence that remains to be fully elucidated experimentally.

Both CT and VWC domains are involved in homo‐ and heterodimerization, as well as in oligomerization. Notably, oligomerization requires an initial dimerization step, likely directed by the CT domain, as previously described in the case of the von Willebrand factor. The VWC domain alone is reported to govern weak dimerization.^1^, ^13^, ^14^, ^15^ Heterotypic and homotypic dimerizations of CCN2 and CCN3 proteins have been reported.^16^ It is therefore assumed that all CCN proteins containing a CT module may exist and act as dimers (reviewed in Monsen VT and Attramadal H.2023,^13^). CCN pathological variants containing the CT module are also expected to act as dimers.^5^

The striking structural conservation of these domains initially led to the assumption that the biological functions of CCN proteins might reflect the sum of the individual activities associated with each domain.^12^ However, it progressively became clear that functional diversity extends beyond simple additive effects.

## 3 MODULARITY AND INTERACTION COMPLEXITY

Despite their structural similarities, CCN proteins regulate opposing cellular processes in both normal and pathological conditions. Notably, experimental evidence indicates that CCN2 and CCN3 are opposing factors in regulating collagen promoter activity.^17^

The multimodular organization of CCN proteins therefore raises fundamental questions regarding the contribution of individual domains to the function of the full‐length protein.

Compilations of ligand–domain interactions indicate that simple binary models of CCN function are insufficient to explain their biological activities. Instead, a more integrated view is required, taking into account ligand diversity, binding affinities, and interaction kinetics.^12^, ^18^, ^19^, ^20^ Such a framework suggests that CCN proteins act as signaling integrators, potentially forming higher‐order complexes that modulate downstream pathways.

Rather than acting independently, these constitutive domains are likely to cooperate, generating emergent properties that cannot be predicted from their isolated functions.^12^, ^13^, ^21^, ^22^ In this context, post‐transcriptional regulatory mechanisms represent an additional layer of complexity that contributes to the diversification of CCN protein functions.^23^

These observations suggest that the C‐terminal domain may play a central role in the formation of higher‐order CCN complexes with regulatory functions that extend beyond the extracellular environment.

## 4 BIOCHEMICAL MODIFICATIONS AND PROTEOLYTIC GENERATION OF TRUNCATED PROTEOFORMS

Post‐translational modifications (PTMs), including phosphorylation, acetylation, and methylation, contribute to protein functional diversity and play essential roles in coordinating signaling networks in both normal and tumor contexts.^24^, ^25^

Proteolytic processing (a form of PTM) has long been recognized as a key post‐translational regulatory mechanism generating biologically active proteoforms.

In addition to biochemical PTM of CCN3 protein by glycosylation,^26^, ^27^ amino‐truncated CCN3 proteoforms containing only the TSP1 and CT domains were identified by microsequencing and the use of domain‐specific antibodies in cellular lysates and culture supernatants of tumor cells.^26^, ^27^, ^28^, ^29^

Truncated CCN2 proteoforms exhibiting distinct biological properties were also identified in pig uterine luminal flushings.^30^

The flexible hinge region separating the N‐terminal (IGFBP and VWC) domains from the C‐terminal (TSP1 and CT) domains is a target for matrix metalloproteinases (MMPs).^31^, ^32^, ^33^ Proteolytic processing of CCN3 at the hinge region separating the two bimodular units has been shown to generate distinct protein fragments. This cleavage effectively dissociates modules that were previously proposed to exhibit opposing transcriptional activities, thereby producing proteoforms with potentially divergent functions.^34^ Such processing events are therefore not merely degradative but may represent a key regulatory step in modulating the functional output and dimerization capacity of the CT module.

The generation by limited proteolysis of these truncated forms suggests that CCN proteins can exist in multiple functional states. Their interactions with different partners across various biological compartments increase their regulatory potential.^13^

The molecular architecture of CCN proteins provides a structural framework for the integration of multiple extracellular signals. In addition, it supports a spatiotemporal model accounting for the complex regulatory mechanisms governing CCN protein expression and activity.^12^

## 5 NUCLEAR CT FUNCTIONS AND TRANSCRIPTIONAL REGULATION

In this section, we examine the transcriptional functions of CCN proteins, with a particular focus on CCN3‐derived amino‐truncated proteoforms and the regulatory role of the C‐terminal (CT) domain. We propose a model in which CT‐dependent dimerization mediates transcriptional repression of specific target genes through interactions with RNA polymerase II.

The first evidence linking CCN3 to tumorigenesis emerged from studies of Myeloblastosis Associated Virus (MAV)‐induced nephroblastomas.^35^, ^36^ Viral insertion within the CCN3 locus led to the expression of an amino‐truncated proteoform lacking the N‐terminal signal peptide. This truncated protein exhibited oncogenic properties and accumulated in the nuclear compartment.^37^

Subsequent experimental approaches demonstrated that the CT domain is both necessary and sufficient to direct nuclear localization. Functional analyses established that the CT domain exerts transcriptional repression, whereas the VWC domain partially counteracts this activity within the full‐length protein.^38^, ^39^

The proteoforms detected in tumor contexts, containing only the TSP1 and CT domains, lack the modulatory influence of the VWC domain and therefore display enhanced CT‐dependent transcriptional repression.

Consistent with this, nuclear CCN3 proteoforms have been observed within transcriptionally active compartments, as demonstrated by their colocalization with transcription‐associated factors such as ICP4,^27^ supporting a direct involvement in nuclear regulatory processes.^33^

At the molecular level, CCN3 has been reported to interact with the Rpb7 subunit of RNA polymerase II, as shown by two‐hybrid and in vitro translation assays.^27^ Although the precise interaction domain has not yet been identified, this observation provides strong evidence for a functional link between CCN3 proteoforms and the core transcriptional machinery. Given the established role of Rpb7 in the regulation of transcription, particularly during pre‐initiation complex (PIC) formation, this interaction suggests a functional link between CCN3 and the regulation of transcriptional initiation.

Taken together, these observations, although obtained in distinct experimental contexts, converge toward a model in which CT‐containing CCN3 proteoforms function as transcriptional regulators within the nuclear compartment.

## 6 CT‐DEPENDENT REPRESSION OF PAI‐2 AND CONSEQUENCES FOR TUMOR PROGRESSION

The convergence of structural, biochemical, and cellular observations described above supports a model in which CT‐containing CCN3 proteoforms function as regulators of gene expression. Although many of these findings were originally reported independently and in different experimental contexts, their integration reveals a coherent and previously underappreciated framework linking CCN3 processing to transcriptional control.

The search for a possible mechanism for regulation of constitutive PAI‐2 (plasminogen activator inhibitor‐2) levels led to the discovery of a new PAI‐2 transcriptional regulatory motif (2400 TRM).^40^, ^41^ Screening of a cell cDNA expression library and mobility shift assays consistently identified a positive clone whose sequence was 100% identical to exon 5 of CCN3, encoding the C‐terminal domain of the protein. The CT protein was shown to specifically bind the 2400 TRM PAI‐2 promoter region, as shown by competitive gel shift analyses.

These experiments provided the first evidence in favor of the involvement of a nuclear CCN3 proteoform in the regulation of PAI‐2 expression and, consequently, plasminogen production.

Although the interaction of the CT domain of CCN3 with the PAI‐2 promoter was identified in earlier studies, its functional significance has remained largely underappreciated and not fully integrated into a broader mechanistic framework.

Given the role of PAI‐2 in regulating plasminogen activation, extracellular matrix remodeling, and tumor‐associated proteolysis,^42^ its transcriptional regulation is expected to have significant consequences for tumor progression, including effects on invasion, migration, and metastasis.

**FIGURE 1:**
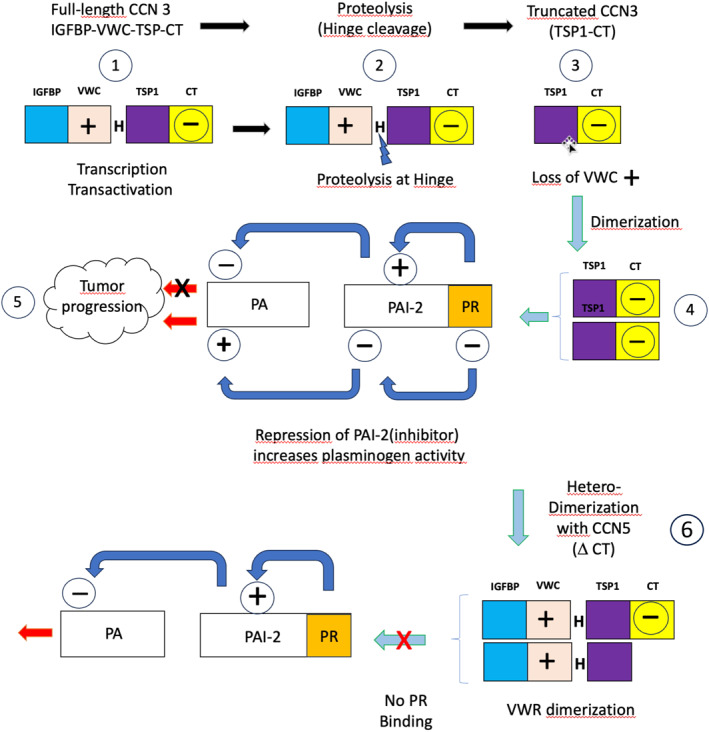
Hypothetical model showing CT‐dependent dimerization of CCN 3. Possible role in transcriptional repression of PAI‐2 and inhibition by CCN5. CCN proteins may form homo‐ and heterodimers via their C‐terminal (CT) domain. Homodimers containing two CT domains could act as transcriptional repressors of the PAI‐2 gene (panels 1–5), whereas heterodimers with CCN5 (lacking the CT domain) would be inactive, resulting in sustained PAI‐2 expression (panel 6). This double negative mechanism would regulate plasminogen activation and downstream biological effects such as fibrinolysis and tumor progression.

Within this framework, CT‐containing CCN3 proteoforms may act as transcriptional repressors of PAI‐2 expression, thereby modulating plasminogen activation in both physiological and pathological contexts (Figure 1).

## 7 CCN5 AS A DOMINANT‐NEGATIVE REGULATOR

Drawing an analogy with the helix–loop–helix (bHLH) transcription factor system,^43^ we propose a working model in which the PAI‐2 expression level, and resulting plasminogen production, could be negatively governed by the interplay of CCN3 and CCN5.

The lack of a CT module in CCN5 would interfere with the ability of CCN3–CCN5 heterodimers to undergo proper CT‐dependent dimerization and to bind DNA targets such as the PAI‐2 promoter.

Within this framework, which remains to be experimentally challenged, homodimers of CCN proteins containing two CT domains would act as transcriptional repressors, whereas heterodimers containing one monomer lacking the CT domain would be structurally incomplete and therefore functionally impaired or inactive (Figure 1).

This model suggests that CCN5 may function as a dominant‐negative regulator of CCN3‐mediated transcriptional repression, thereby introducing an additional level of regulation in the control of plasminogen activation pathways. Such a mechanism would provide a conceptual basis for the functional divergence observed between CCN family members and further supports the central role of the CT domain in mediating transcriptional regulation.

## 8 CONCLUSION AND PERSPECTIVES

In several cases, the production of plasminogen activators has been reported to correlate with malignancy.^44^, ^45^, ^46^, ^47^ Conversely, high levels of PAI‐2 decrease tumor growth and metastasis.^48^ These features align with the biological activities of nuclear CCN3 proteoforms, whose generation under normal and pathological conditions remains to be fully elucidated.

The rationale of our working model is based on the identification of CCN3 as a transcription factor (or co‐factor) interacting with the central transcriptional machinery.

First, in the full‐length CCN3 protein, the coexistence of modules with opposing transcriptional activities (governed by the VWC positive and CT negative domains) suggests that regulatory balance is an intrinsic property of the protein. Proteolytic cleavage at the hinge region separates these activities into distinct entities, generating amino‐truncated proteoforms enriched in TSP1 and CT modules. These proteoforms, through the CT domain, acquire the ability to translocate to the nucleus and localize within transcriptional compartments.

This is the first pillar of our reasoning.

The second pillar is the reported interaction between CCN3 and the Rpb7 subunit of RNA polymerase II. It further supports a direct involvement in transcriptional regulation, particularly at the level of pre‐initiation complex (PIC) assembly. In this context, we propose that the CT domain of CCN3 acts as a transcriptional cofactor contributing to the positioning or stabilization of the pre‐initiation complex at specific promoters.

The third pillar is the dimerization of CCN proteins mediated by the C‐terminal domain. The identification of homo‐ and heterodimers of CCN2 and CCN3 confirms that CCN proteins containing a CT module engage in dimerization, which represents the first step toward multimerization of the four constitutive domains.

The fourth pillar relates to target gene regulation. Applied to genes such as PAI‐2, this model suggests that CT‐containing CCN3 proteoforms may facilitate promoter engagement by the transcriptional machinery. Furthermore, given the involvement of the CT module in early steps of CCN3 dimerization, it is conceivable that dimer formation of N‐terminally truncated proteoforms represents a regulatory step controlling pre‐initiation complex positioning and transcriptional activity.

Altogether, this sequence of events provides a working mechanistic framework linking CCN3 proteolytic processing, nuclear localization, dimerization, and transcriptional regulation, with potential implications for the control of plasminogen‐related pathways in both physiological and tumorigenic contexts.

This model is based on both established and hypothetical data.

Future experimental approaches should allow the scientific community to validate or refute the proposed model.

## AUTHOR CONTRIBUTIONS

The author solely contributed to the conception and writing of this review.

## CONFLICT OF INTEREST STATEMENT

The authors declare no conflicts of interest.

## ETHICS STATEMENT

No ethic, nor patient statement.

## Data Availability

Data sharing not applicable to this article as no datasets were generated or analyzed during the current study.
